# Comparison Between Skeletally‐Anchored and Conventional Maxillary Protraction in Growing Class ΙΙΙ Patients: A Systematic Review and Meta‐Analysis

**DOI:** 10.1002/cre2.70466

**Published:** 2026-09-27

**Authors:** Christianna Iris Papadopoulou, Nusha Paschaei, Giulia Brunello, Rebecca Jungbauer, Kathrin Becker

**Affiliations:** ^1^ Department of Orthodontics and Dentofacial Orthopedics Center for Oral Health Sciences CC3, Charité Universitätsmedizin Berlin, Corporate Member of Freie Universität Berlin and Humboldt‐Universität Berlin Berlin Germany; ^2^ Department of Oral Surgery University Hospital of Düsseldorf Düsseldorf Germany; ^3^ Department of Orthodontics University Medical Centre Regensburg Regensburg Germany

**Keywords:** class III, facemask, maxillary protraction, skeletal anchorage

## Abstract

**Objective:**

The aim of this systematic review was to evaluate the skeletal and dental effects of skeletally‐anchored (SA) maxillary protraction compared with conventional facemask (FM) therapy in growing patients with Class III malocclusion, while controlling for the confounding effect of rapid maxillary expansion (RME).

**Materials and Methods:**

A systematic literature search (MEDLINE, Cochrane Library, Virtual Health Library, Web of Science, Google Scholar), which involved two screening stages, was performed until January 2026 and identified prospective clinical trials comparing SA with conventional tooth‐borne FM therapy. Only studies in which expansion protocols were applied uniformly across groups were included. Risk of bias was assessed with RoB 2 and ROBINS‐I tools, and random effects meta‐analyses were conducted for cephalometric outcomes. The certainty of the evidence was evaluated according to the grading of recommendations, assessment, development, and evaluation (GRADE) approach.

**Results:**

Five prospective trials (3 RCTs, 2 CCTs; *n* = 194 participants) met the inclusion criteria. Random‐effects meta‐analyses showed a modest but significant greater increase in SNA for SA (MD 0.65°; 95% CI 0.18–1.13, *I* ^2^ = 0.0%) and consistently fewer dental side effects with SA: upper‐molar mesialization (−1.14 mm; −2.12 to −0.17, *I* ^2^ = 10.3%), molar extrusion (−0.74 mm; −1.07 to −0.40, *I* ^2^ = 0.0%), and maxillary incisor proclination (−2.47°; −4.14 to −0.80, *I* ^2^ = 0.0%). The mandibular plane rotation did not differ significantly (−0.38°; −1.67 to 0.91, *I* ^2^ = 45.6%). Risk of bias was assessed as “some concerns” in RCTs and “serious” in non‐randomized studies. The certainty of evidence ranged from very low to moderate according to GRADE.

**Conclusions:**

Within the limitations of the available short‐term evidence, and given the low‐to‐moderate certainty of the pooled estimates, SA was associated with fewer dental side effects and a statistically significant, though not clinically relevant, gain in skeletal efficacy. Future long‐term, age‐stratified randomized trials are needed to confirm the long‐term stability of these findings.

## 1 Introduction

Managing skeletal Class III malocclusion remains a significant orthodontic challenge. With an estimated prevalence of 5.93% in the permanent dentition (Alhammadi et al. 2018; Zere et al. 2018), it is typically caused by maxillary deficiency, mandibular prognathism, or a combination of both (Solano‐Mendoza et al. 2012; Toffol et al. 2008; Zere et al. 2018). Multiple treatment options have been suggested, such as early maxillary orthopedic protraction, orthodontic camouflage, or orthognathic surgery in fully grown patients (Delaire 1971; Hickham 1991; Jäger et al. 2001; Mandall et al. 2010). The treatment of maxillary hypoplasia is supposed to be most effective during the early mixed dentition phase (Baccetti et al. 2000, 1998; Jäger et al. 2001; Ngan and Moon 2015; Ngan et al. 1997). However, it has been reported that maxillary protraction is associated with undesirable side effects, such as mesialization of posterior teeth, incisor proclination, bite opening due to posterior tooth extrusion, and clockwise mandibular rotation (Moon et al. 2015; Ngan et al. 2015, 1997; Williams et al. 1997). To overcome the limitations of conventional facemask (FM) treatment with dental anchorage, maxillary protraction with skeletal anchorage (SA) has been proposed. Temporary anchorage devices (TADs), such as miniplates (MP) or orthodontic mini‐implants (OMIs) combined with FM, or miniplates placed in the mandible with intermaxillary elastics (Kircelli and Pektas 2008), have been shown to deliver purely orthopedic forces and reduce dental side effects (Ge et al. 2012; Kircelli et al. 2006; Maino et al. 2018).

In recent years, various bone‐anchored maxillary protraction methods have been described, including the combination of an FM with OMIs placed in the zygomaticoalveolar crest or the anterior palate. Alternatively, purely bone‐anchored protraction can be achieved using miniplates in the zygomatic or lateral piriform region of the maxilla and in the mandible, with intermaxillary elastics. In this approach, the resulting force vector is supposed to be directed closer to the center of resistance of the nasomaxillary complex, resulting in a shorter lever arm, and therefore a reduced clockwise rotation of the palatal and mandibular planes (De Clerck et al. 2009; Elnagar et al. 2016; Ge et al. 2012; Kircelli et al. 2006; Maino et al. 2018; Ngan et al. 2015; Nienkemper et al. 2015, 2013; Solano‐Mendoza et al. 2012; Wilmes et al. 2010).

Rapid maxillary expansion (RME) is often combined with facemask treatment. Liou et al. (2005) and Liou and Tsai (2005) introduced a modification of RME called Alternate Rapid Maxillary Expansion and Constriction (Alt‐RAMEC), based on the idea that alternating expansion and constriction further disarticulates the circummaxillary sutures. According to previous research, RME or Alt‐RAMEC prior to maxillary protraction “loosens” the mid‐palatal and most likely the circummaxillary sutures, resulting in greater maxillary advancement (Baik 1995; Gallagher et al. 1998; Haas 1973; Isci et al. 2010; Wertz 1970). However, some studies found no significant cephalometric differences between patients treated with FM alone versus FM/RME (Tortop et al. 2007; Vaughn et al. 2005). Previous systematic reviews have compared conventional versus bone‐anchored maxillary protraction protocols, however, none have controlled for the potential confounding factor of prior RME. Because RME can influence skeletal outcomes (Baik 1995; Gautam et al. 2009; Jäger et al. 2001; Kilic et al. 2008; Kim et al. 1999; Liou 2005; Liou and Tsai 2005), excluding studies that did not apply a uniform RME protocol between the compared groups increases methodological homogeneity and allows a more isolated evaluation of the effect of skeletal anchorage itself.

The objective was to compare short‐term dental anchorage loss (molar mesialization, molar extrusion, incisor proclination) and skeletal effects (SNA, WITS appraisal, SN‐MnPl) between skeletal anchorage and conventional facemask protraction in growing Class III patients, restricting inclusion to trials with uniform expansion protocols to reduce confounding by RME/Alt‐RAMEC.

## 2 Material and Methods

This systematic review was structured and conducted according to the Preferred Reporting Items for Systematic Reviews and Meta‐Analyses (PRISMA) (Page et al. 2021). The review protocol was created and registered at the PROSPERO database (CRD42024622038).

### 2.1 PICOS

The study design and formulation of the research question followed the PICOS model, as shown in Table 1.

**Table 1:** Study inclusion and exclusion criteria (PICOS).

Field	Inclusion	Exclusion
Patients (P)	Patients requiring early Class III treatment (all ethnicities)	– Animal studies
		– *In vitro* studies
		– Patients > 15 years old
		– Studies with fewer than five patients included
		– Patients with systemic diseases, periodontal disease, or craniofacial syndromes
Intervention (I)	– Bone‐anchored maxillary protraction (miniplates, orthodontic mini‐implants)	– Studies including orthognathic surgery
	– Conventional treatments with dental anchorage	– Studies in which RME was performed in only one treatment group (either skeletal group or conventional treatment group)
		– Studies where the control group's treatment was not specified
Comparison (C)	Bone‐anchored maxillary protraction in comparison to conventional treatment methods:	
	– Skeletal changes: changes in WITS, SNA, mandibular plane angle (SN‐MnPl)	
	– Changes in anchorage loss: (upper first molar mesialization and extrusion, upper incisor proclination)	
	– Treatment duration	
	– Associated risks	
Outcome (O)	Qualitative and quantitative analysis of skeletal changes (SNA, WITS, SN‐MnPl), anchorage loss (molar mesialization and extrusion, incisor proclination), treatment duration and associated risks.	Lack of clinical data on anchorage loss.
Study design (S)	Prospective CCTs or RCTs will be included.	Case reports, systematic reviews, meta‐analyses, reviews, non‐randomized or retrospective controlled clinical trials, cohort studies and case‐control studies.

Abbreviations: CCT, controlled clinical trials; RCT, randomized clinical trials; RME, rapid maxillary expansion; SN‐MnPl, Sella–Nasion–Mandibular plane.

### 2.2 Eligibility Criteria

The methodology and eligibility criteria were established before conducting the systematic review. Any deviations from the initial research protocol were documented. Studies were screened and evaluated according to the following inclusion and exclusion criteria:

Inclusion criteria

– English language
– Prospective controlled clinical trials (CCTs) or randomized controlled clinical trials (RCTs) in humans comparing a “skeletal anchorage treatment group” with a “conventional treatment group”
– Patients of the general population (all ethnicities, community‐dwelling)

Exclusion criteria:

– Studies including orthognathic surgery instead of maxillary protraction
– Patients > 15 years old
– Inclusion of less than five patients
– Previous orthodontic treatment
– Inclusion of patients with systemic diseases, periodontal disease, craniofacial syndromes or abnormalities
– Undefined control group treatment
– Lack of clinical data on anchorage loss
– RME performed only in one treatment group

The primary review question was to assess the efficacy of skeletally‐anchored maxillary protraction compared to conventional treatment approaches in growing patients requiring Class III treatment.

### 2.3 Search Strategy

An electronic search of five databases (MEDLINE, Cochrane Library, Virtual Health Library, Web of Science, Google Scholar) was performed until January 2026, using the following search strategy for each database: (“class III” OR “early class III” OR “class III treatment” OR “maxillary protraction”) AND (“mini implants” OR “miniscrews” OR “orthodontic mini implants” OR “skeletal anchorage” OR “palatal implant” OR “skeletal” OR “skeletal anchorage” OR “temporary anchorage device” OR “TAD” OR “bone screw”) AND (“anchorage loss” OR “efficacy” OR “side effects” OR “effect” OR “treatment outcome”).

A manual hand search of the available literature was also conducted. The reference lists of the included studies were manually reviewed for additional relevant studies.

### 2.4 Study Selection

The study selection process involved two stages of screening. Two review authors (C.I.P. and N.P.) independently screened the titles and abstracts of the identified studies and reports. Inter‐reviewer agreement was assessed by means of Cohen's Kappa coefficient. They were not blinded to the identity of the authors, their institutional affiliations, and research findings. Irrelevant reports were excluded, while full‐text articles were assessed for eligibility based on predefined criteria. The search results were imported into Endnote (Clarivate Analytics, PA, USA) for further selection. During the first stage, the titles and abstracts were screened and evaluated according to predefined inclusion criteria. Studies meeting eligibility criteria or lacking sufficient information were kept for full‐text assessment.

At the second stage of selection, all full‐text articles of potentially relevant studies identified during the first stage were examined. During this procedure, the pre‐selected publications were evaluated against the predefined exclusion criteria. Any disagreements regarding article inclusion during the first and second stage of study selection were resolved through discussion with the senior author (K.B.).

### 2.5 Data Collection

The same authors (C.I.P. and N.P.) independently performed data extraction in duplicate. Any disagreements were again resolved through discussion with the senior author (K.B.). A data extraction template was created including the study ID, study design, patient number, sex distribution, age, type of skeletal anchorage, number of OMIs or MPs, location of TADs, time points of observation, treatment duration, control intervention, measurement method, and primary and secondary outcomes.

### 2.6 Risk of Bias Assessment

Methodological quality was assessed in two different phases. In the first phase, it was conducted independently by two authors (C.I.P. and N.P.) based on the included full‐text articles. In the second phase, disagreements were resolved through consultation with the senior author (K.B.) using the appropriate risk of bias assessment tools. The revised Cochrane risk‐of‐bias tool for RCTs (RoB‐2) was used to assess the risk of bias of the RCTs (Sterne et al. 2019), and the risk‐of‐bias in non‐randomized studies of interventions (ROBINS‐I) tool was used to assess the risk of bias of the non‐randomized studies (Sterne et al. 2016). When encountering missing data or zero values, the corresponding authors of the published articles were contacted as needed.

### 2.7 Reporting Bias Assessment

Publication bias was assessed visually using funnel plots for outcomes included in the meta‐analysis. Asymmetry in funnel plots was considered indicative of potential reporting or publication bias.

### 2.8 Unit of Analysis and Missing Data Issues

The unit of analysis was the individual patient. For Liang et al. (2021), left and right upper first molar values were combined into a single bilateral mean and standard deviation, assuming a within‐patient left‐right correlation of *r* = 0.80, consistent with the high symmetry typically observed in bilateral dental measurements. When studies reported outcomes as medians and interquartile ranges (IQRs) instead of means and standard deviations, the median and IQR values at *T*_0_ and *T*_1_ were exported and converted to mean (SD) at each time point using established estimators for sample statistics from quantiles (Luo et al. 2018; Wan et al. 2014):

Equation 1. Estimated mean and standard deviation from median and IQR

(1) $$x®\;\approx \;\frac{Q_{1}+\mathrm{median}+Q_{3}}{3},\;\mathrm{SD}\;\approx \;\frac{Q_{3}-Q_{1}}{1.35}$$

Then, the change score (Δ = *T*_1_ − *T*_0_) and its SD was calculated via

Equation 2. Standard deviation of the change score (*r* = correlation between *T*_0_ and *T*_1_)

(2) $${\mathrm{SD}}_{\Delta }=\sqrt{{{\mathrm{SD}}_{T0}}^{2}+{{\mathrm{SD}}_{T1}}^{2}-2r\;{\mathrm{SD}}_{T0}{\mathrm{SD}}_{T1}}$$

assuming a within‐person correlation *r* = 0.50.

### 2.9 Effect Measures and Synthesis Methods

The statistical analysis was performed using R (v.4.4.2; R Core Team) and RStudio (Posit Software, PBC, Boston, MA, USA), primarily with the meta package (v.8.2‐1). Data handling and preparation used the openxlsx, dplyr, and stringr packages.

Random‐effects meta‐analyses were fitted using restricted maximum likelihood (REML) estimation of between‐study variance, with Hartung–Knapp‐adjusted 95% confidence intervals. Analyses evaluated differences between skeletal and conventional maxillary protraction protocols across dental outcomes (upper molar mesialization and extrusion, upper incisor proclination relative to the palatal plane) and skeletal outcomes (WITS appraisal, SN‐MnPl angle, SNA angle). Effects were calculated as skeletal anchorage minus conventional facemask. Heterogeneity was assessed for each outcome. A sensitivity analysis restricted to randomized controlled trials was performed to assess robustness to study design, with an additional analysis excluding Alzoubi et al. (2023) given its reconstructed data. Funnel plots were used to assess publication bias. Statistical significance was set at *p* < 0.05.

### 2.10 Heterogeneity Assessment

Statistical heterogeneity among studies was evaluated using standard heterogeneity statistics (*I* ^2^ and corresponding *χ* ^2^ tests). The random‐effects model was selected a priori due to expected clinical and methodological variability among included studies.

### 2.11 Certainty Assessment (GRADE)

The certainty of the evidence for each outcome was assessed using the Grading of Recommendations Assessment, Development and Evaluation (GRADE) approach (Guyatt et al. 2013). Evidence was evaluated across the domains of risk of bias, inconsistency, indirectness, imprecision, and publication bias. The overall certainty of evidence was categorized as high, moderate, low, or very low. The GRADEpro GDT was used to evaluate the evidence and to get the “summary of findings table.”

## 3 Results

### 3.1 Study Selection

**Figure 1:**
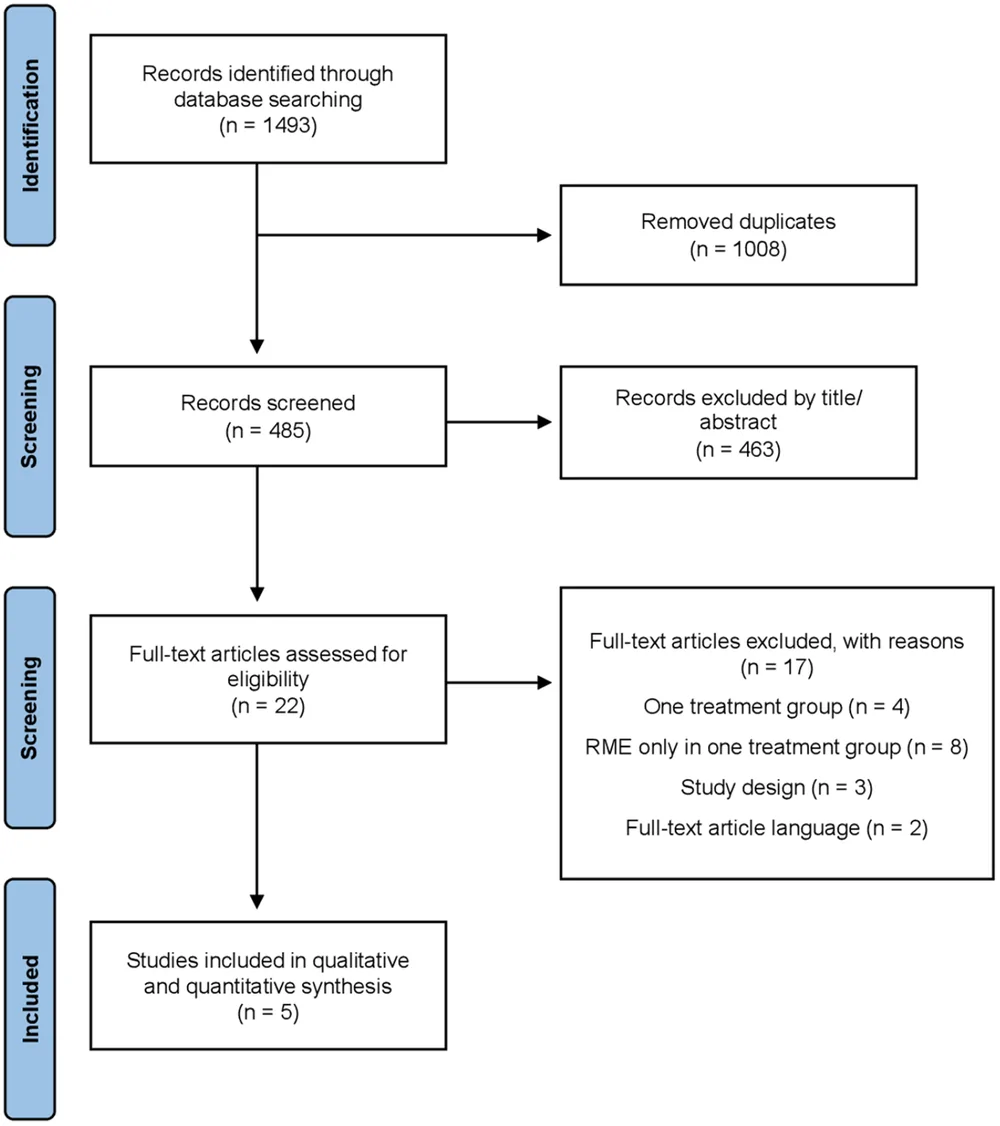
Flow diagram for the selection of studies according to PRISMA.

A total of 1493 potentially relevant titles and abstracts were generated through electronic and manual searches. After screening for duplicates and applying the inclusion criteria to titles and abstracts, 22 articles were found eligible for full‐text assessment. In the second phase, 17 of these 22 studies were excluded, as they did not meet the inclusion criteria of this systematic review (Table 2). Finally, five articles were included in the qualitative and quantitative synthesis (Ağlarcı et al. 2016; Alzoubi et al. 2023; Liang et al. 2021; Şar et al. 2011; Seiryu et al. 2020). Inter‐reviewer agreement was high, with kappa values of 0.98 for title/abstract screening and 0.88 for full‐text selection. The flow chart for the various steps in the search is presented in Figure 1.

**Table 2:** Excluded studies in phase II (with reasons).

Excluded studies	Reason
*N* = 1	No skeletal anchorage group
Celikoglu et al. (2015)	
*N* = 3	RME only in one group, retrospective study
Hino et al. (2013); Lee et al. (2020); Tabellion and Lisson (2024)	
*N* = 5	RME only in one group
de Souza et al. (2019); Ge et al. (2012); Jang et al. (2021); Lee et al. (2012), (2022)	
*N* = 2	Article in Chinese
Ma et al. (2016); Ziyu et al. (2024)	
*N* = 3	Retrospective study
Ngan et al. (2015); Buyukcavus et al. (2020); Jamilian et al. (2011)	
*N* = 3	Only one treatment group, untreated control group
De Clerck et al. (2010); Kamel et al. (2023); Mandall et al. (2024)	

Depending on data availability, four trials contributed to most meta‐analyses; Alzoubi et al. (2023) contributed only to SNA, U1‐PP, and WITS after reconstruction of mean/SD from medians/IQRs. For the WITS appraisal, Liang et al. (2021) and Seiryu et al. (2020) were excluded since the data were not presented. Since Alzoubi et al. (2023) reported median values and interquartile ranges, mean values and standard deviations were derived as described in the methods section. The numerical outcome data (means and standard deviations) of all studies are presented in Table S1.

### 3.2 Included Study Characteristics

Of the selected studies, three were randomized controlled clinical trials (Alzoubi et al. 2023; Liang et al. 2021; Seiryu et al. 2020). All studies were published between 2011 and 2023. The descriptive characteristics of the included studies are outlined in Table 3. They compared a “skeletal anchorage treatment group” with a “conventional treatment group,” with three studies performing maxillary expansion in all subjects before maxillary protraction (Alzoubi et al. 2023; Liang et al. 2021; Şar et al. 2011).

**Table 3:** Characteristics of the included studies.

Reference	No. of participants	Type of study	Control intervention	Location (number) of TADs	WITS (difference T1–T0, mean value [SD])	U6 Anchorage loss (difference in molar mesialization, T1–T0, mm)
Aglarci et al. (2016)	50 (25 SA; 25 FM)	CCT	FM	UJ (*n* = 2): between second premolar and first molars LJ (*n* = 2): between the mandibular lateral incisors and canines	SA:3.87 (SD 2.55) FM:5.03 (SD 2.23)	SA:0.58 (SD 1.32) FM:1.69 (SD 1.72)
Şar et al. (2011)	45 (15 SA/FM/RME; 15 FM/RME; 15 untreated)	CCT	FM/RME	UJ (*n* = 2): lateral to the apertura piriformis region of the maxilla	SA/FM/RME: 5.43 (SD 1.69) FM/RME: 6.56 (SD 2.88) Control: −0.53 (SD 1.14)	SA/RME: −0.03 (SD 0.63) FM/RME: 1.93 (SD 2.35) Control: −0.20 (SD 0.64)
Alzoubi et al. (2023)	34 (17 SA/FM/RME; 17 FM/RME)	RCT	FM/RME	UJ (*n* = 2): paramedian	SA/FM/RME: 3.20 (SD 2.86) FM/RME: 4.70 (SD 3.22)	—
Liang et al. (2021)	41 (20 SA/FM/RME; 21 FM/RME)	RCT	FM/RME	UJ (*n* = 2): in the anterior segment on both sides of the maxilla	—	SA/FM/RME: U6L: 4.88 (SD 3.15) U6R: 4.66 (SD 2.25) FM/RME: U6L: 6.05 (SD 2.28) U6R: 5.82 (SD 2.57)
Seiryu et al. (2020)	39 (19 SA/FM; 20 FM)	RCT	FM	UJ (*n* = 1): anterior region of the palate	—	SA/FM: 2.60 (SD 2.10) FM: 2.90 (SD 2.00)

Abbreviations: CCT, prospective controlled clinical trial; FM, facemask; LJ, lower jaw; NS, not significant; RCT, randomized controlled clinical trial; RME, rapid maxillary expansion; SA, skeletal anchorage; SD, standard deviation; T0, before treatment; T1, after treatment; TAD, temporary anchorage device; U6, upper first molar; UJ, upper jaw.

The inclusion criteria of the selected studies focused on patients with skeletal Class III malocclusion, primarily characterized by maxillary deficiency and/or mandibular protrusion (ANB < 0° or WITS appraisal < −2 mm) (Ağlarcı et al. 2016; Şar et al. 2011). All studies included patients with an anterior crossbite, retrusive nasomaxillary complex, and a concave facial profile. Regarding age and growth stage, most studies selected prepubertal or pubertal patients, assessed through Cervical Vertebral Maturation (CVM) stages or hand‐wrist radiographs (Ağlarcı et al. 2016; Alzoubi et al. 2023; Liang et al. 2021; Seiryu et al. 2020). The age range of participants varied between 8 and 12 years. Patients were required to have normal or increased overbite, an Angle Class III molar relationship, and no previous orthodontic treatment.

### 3.3 Risk of Bias in Included Studies

**Figure 2:**
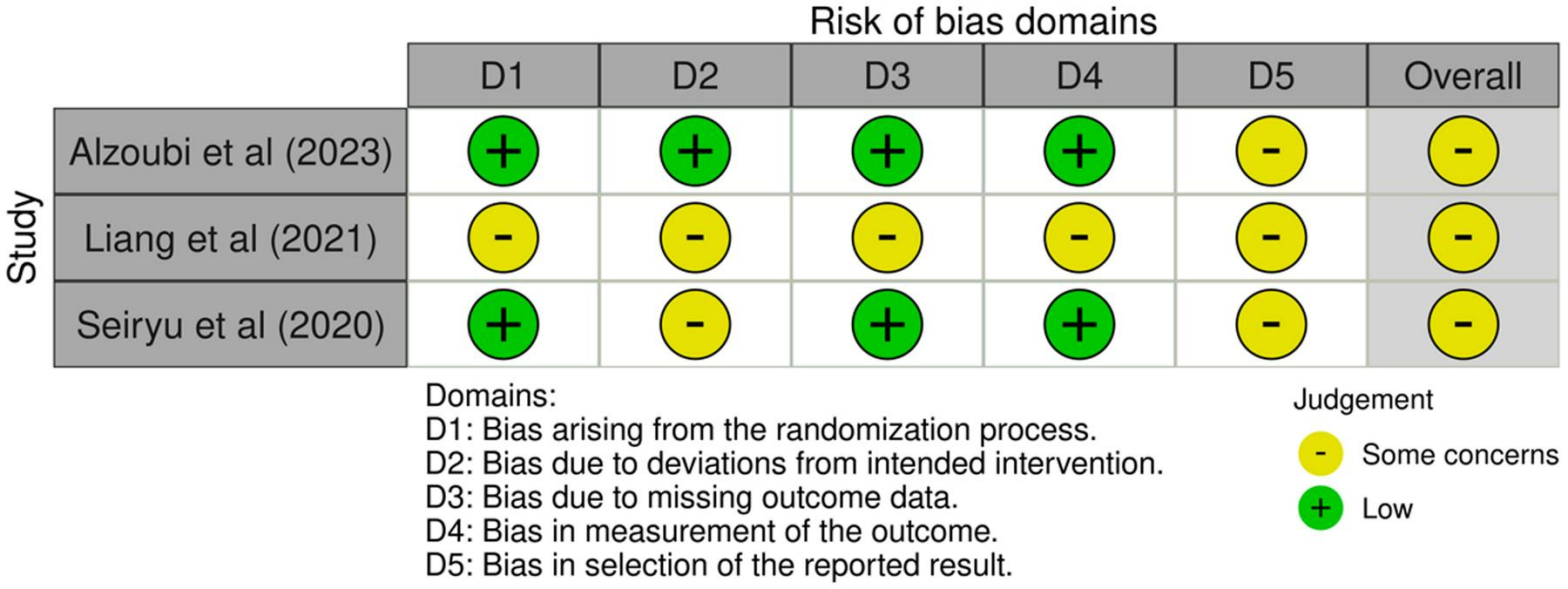
Risk of bias domains for randomized controlled trials, as assessed with the RoB−2 tool.

**Figure 3:**
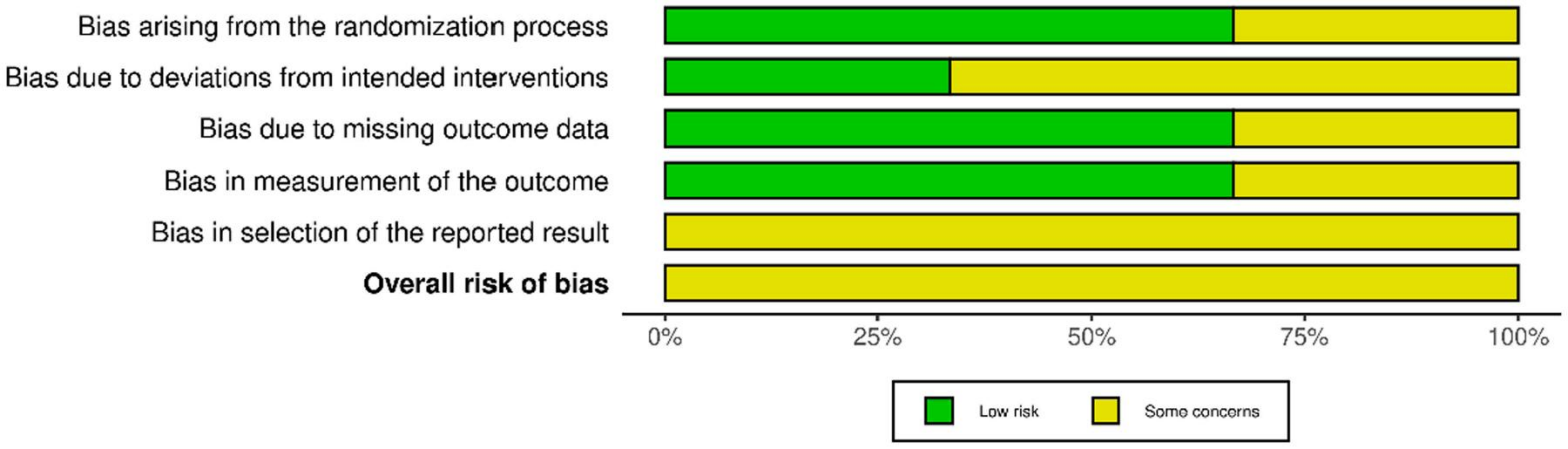
Collective data for risk of bias randomized controlled trials according to each domain.

**Figure 4:**
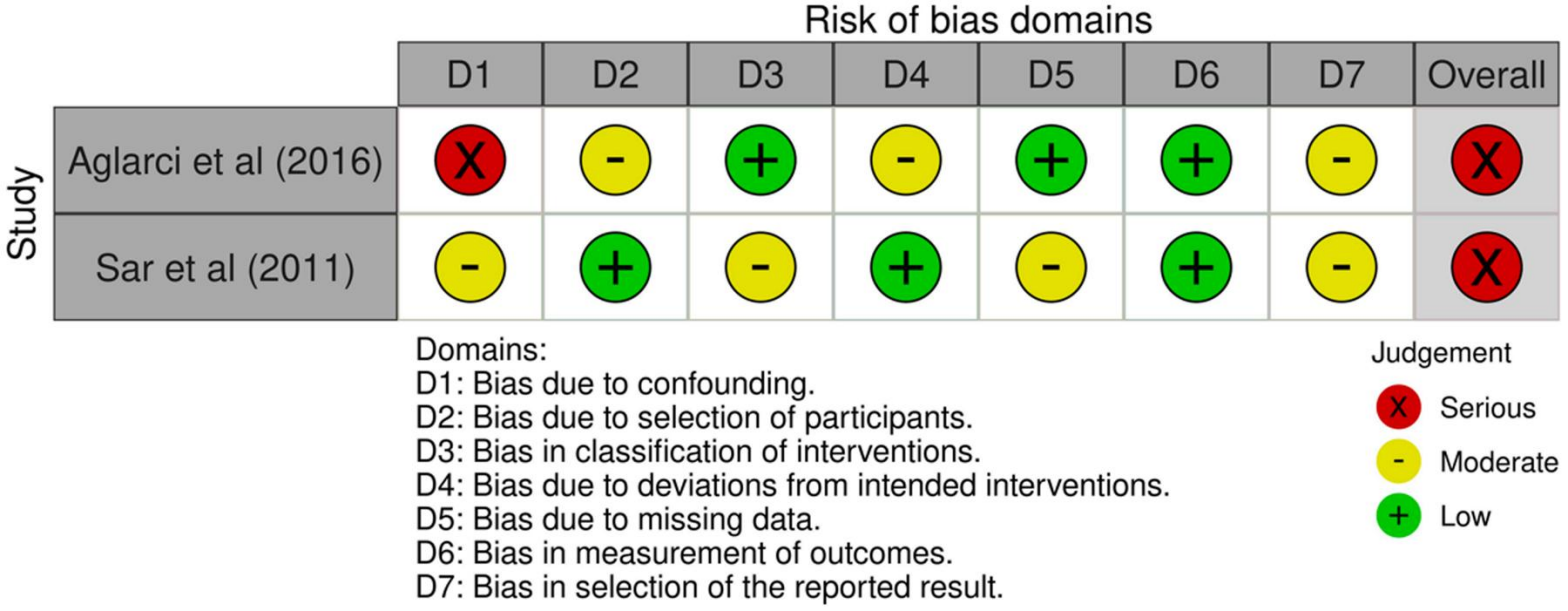
Risk of bias domains for non‐randomized studies, as assessed with the ROBINS‐I tool.

**Figure 5:**
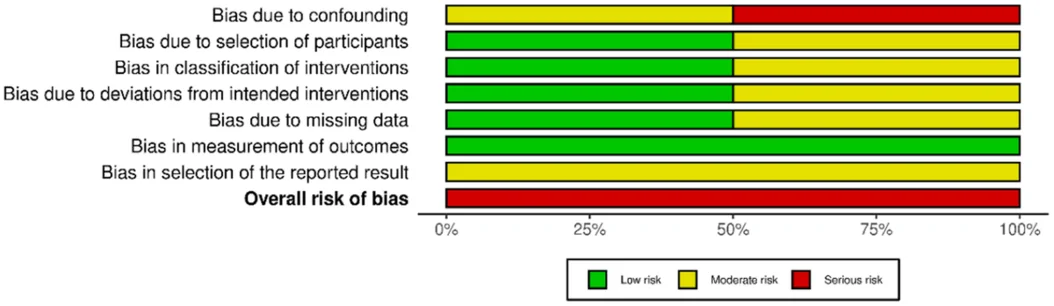
Collective data for risk of bias of non‐randomized studies according to each domain.

The reviewers' risk of bias assessment for each included RCT is summarized in Figures 2 and 3. All RCTs were assessed as having “some concerns.” Among the two non‐randomized studies, both presented a “serious” risk of bias (Figures 4 and 5). Although lack of blinding of participants and clinicians was observed across all studies, this limitation is inherent to orthodontic interventions and was not considered the primary source of bias. In the randomized trials, “some concerns” were mainly related to insufficient reporting of the randomization process and allocation concealment, which may introduce selection bias. In the non‐randomized studies, the “serious” risk of bias was primarily driven by confounding and selection bias. Baseline comparability between groups was not consistently ensured, and adjustments for potential confounders were either insufficient or not clearly reported. Given the observational design, these factors may have influenced the estimated treatment effects. No studies were excluded based on the risk of bias assessment; however, the identified methodological limitations, particularly in non‐randomized studies, should be considered when interpreting the results of this review.

### 3.4 Description of Individual Studies

All studies compared a bone‐anchored maxillary protraction group with a conventional treatment group. Aglarci et al. used two OMIs (1.6 × 10 mm) on the upper jaw inserted between the maxillary second premolars and molars and two I‐shaped titanium miniplates on the lower jaw placed between the mandibular lateral incisors and canines (Ağlarcı et al. 2016). A bite plate was inserted into the upper arch, and Class III elastics were applied with a force of 200 g between each miniplate and OMI.

The rest of the included studies used a bone‐anchored facemask for maxillary protraction. For the skeletal anchorage of the facemask, either miniplates were surgically placed laterally to the piriform aperture of the maxilla (Şar et al. 2011), or OMIs were used, positioned in different locations, such as the anterior palate (Seiryu et al. 2020), paramedian palate (Alzoubi et al. 2023), or in the anterior segment on both sides of the maxilla (Liang et al. 2021). Protraction was achieved using Class III elastics, with loading ranging from 200 g (Ağlarcı et al. 2016) to 500 g (Alzoubi et al. 2023), applied between one (Şar et al. 2011) and 4 weeks (Liang et al. 2021; Seiryu et al. 2020) after implant placement.

Three studies performed RME before maxillary protraction with a bone‐anchored facemask using Hyrax expanders in both treatment groups (Alzoubi et al. 2023; Liang et al. 2021; Şar et al. 2011). One study used a variation of RME, that is, Alternate Rapid Maxillary Expansion and Constriction (Alt‐RAMEC), with 7 weeks of twice‐daily alternate opening and closing cycles to further disarticulate the circummaxillary sutures and subsequently enhance forward maxillary translation (Alzoubi et al. 2023).

### 3.5 Heterogeneity and Sensitivity Analysis

**Figure 6:**
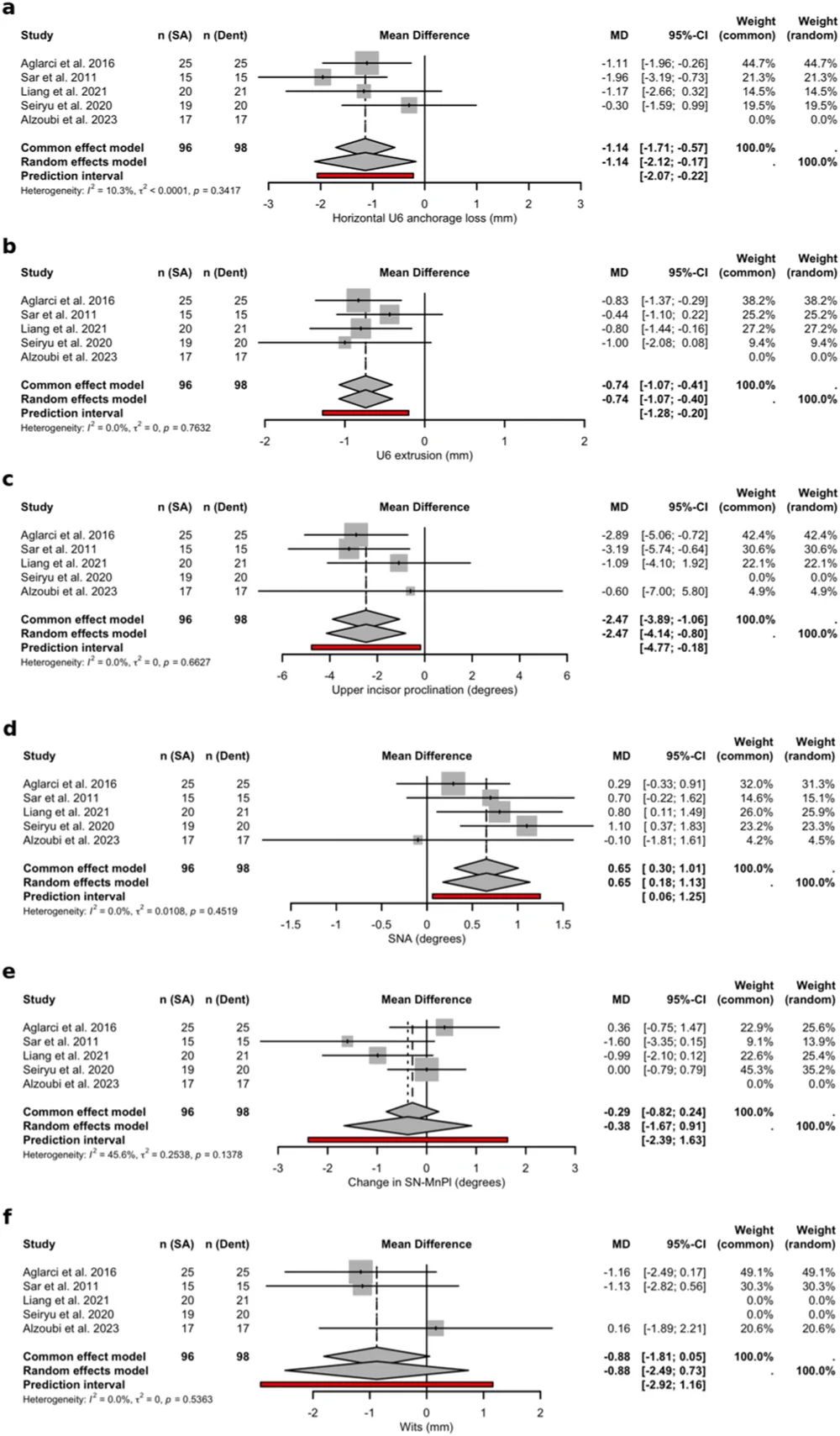
Forest plots (a–f) for molar mesialization, molar extrusion, incisor proclination, SNA, changes in mandibular plane, and WITS appraisal.

The results showed low heterogeneity between the studies (*I* ^2^ = 0%–45.6%). Given the small number of studies contributing to individual outcomes, these estimates should be interpreted cautiously, as low *I* ^2^ values may reflect limited power to detect heterogeneity. The results of the primary meta‐analyses are presented in Figure 6a–f.

Sensitivity analyses restricted to RCTs (Analysis A: all available RCTs, including Alzoubi et al. 2023 where reported; Analysis B: excluding Alzoubi et al. given its reconstructed data) showed point estimates consistent in direction with the primary analyses, but with substantially wider confidence intervals. None of the four poolable outcomes (U6 anchorage loss, U6 extrusion, SNA, SN‐MnPl), including three that were significant in the primary analysis, remained statistically significant in either analysis. This indicates substantial imprecision when restricting the evidence base to RCTs alone, likely reflecting the small number of available trials rather than a true absence of effect (Figures S1 and S2).

### 3.6 Reporting Biases

Funnel plots were inspected to assess potential publication bias and did not indicate clear asymmetry (Figure S3). However, interpretation of funnel plots is limited by the small number of included studies, which reduces their sensitivity and reliability in detecting true asymmetry. Therefore, the possibility of publication bias, small‐study effects, or selective reporting cannot be excluded and should be considered when interpreting the findings.

### 3.7 Certainty of Evidence (GRADE)

The certainty of evidence assessed using the GRADE approach ranged from very low to moderate across outcomes. Evidence was downgraded primarily due to risk of bias in non‐randomized studies and imprecision related to small sample sizes. Evidence for dental anchorage outcomes was rated as low certainty, whereas skeletal outcomes were rated as low to moderate certainty due to variability in study designs and outcome reporting (Table 4).

**Table 4:** Summary of findings according to GRADE.

Summary of findings for: Comparison between bone‐anchored and conventional maxillary protraction in growing Class ΙΙΙ patients: A systematic review and meta‐analysis.							
Outcomes	No. of participants (studies)	Relative effect (95% CI)	Absolute effects (95% CI)			Certainty of the evidence (GRADE)	Comments
			Conventional maxillary protraction	Bone‐anchored maxillary protraction	Difference		
Upper molar mesialization (mm)	160 (4 studies)	—	0	—	−1.14 (−2.12 to −0.17)	⊕⊕◯◯ Low^a^, ^b^	Skeletal anchorage results in less mesial movement (less anchorage loss) of the upper molars compared with conventional facemask therapy.
Upper molar extrusion (mm)	160 (4 studies)	—	0	—	−0.74 (−1.07 to −0.40)	⊕⊕⊕◯ Moderate^a^	Skeletal anchorage results in less extrusion of the upper molars compared with conventional facemask therapy.
Maxillary incisor proclination (degrees)	155 (4 studies)	—	0	—	−2.47 (−4.14 to −0.80)	⊕⊕◯◯ Low^a^, ^b^	Skeletal anchorage results in less proclination of the maxillary incisors compared with conventional facemask therapy.
SNA angle (degrees)	194 (5 studies)	—	0	—	0.65 (0.18 to 1.13)	⊕⊕⊕◯ Moderate^a^	Skeletal anchorage may result in a slightly greater increase in SNA angle compared with conventional facemask therapy.
Mandibular plane angle (SN–MnPl), (degrees)	160 (4 studies)	—	0	—	−0.38 (−1.67 to 0.91)	⊕◯◯◯ Very Low^a^, ^b^, ^c^	There may be little to no difference in mandibular plane angle between skeletal anchorage and conventional facemask therapy.
WITS appraisal (mm)	114 (3 studies)	—	0	—	−0.88 (−2.49 to 0.73)	⊕⊕◯◯ Low^a^, ^b^	There may be little to no difference in WITS appraisal between skeletal anchorage and conventional facemask therapy.

Abbreviations: CI, confidence interval; MD, mean difference.

^a^ Downgraded due to methodological limitations and lack of blinding due to intervention type.

^b^ Downgraded due to limited sample size and wide confidence intervals.

^c^ Downgraded due to moderate heterogeneity between studies.

### 3.8 Synthesis and Intervention Effects

Overall, skeletal anchorage protocols showed reduced dental side effects compared with conventional facemask therapy, while skeletal outcomes showed modest improvements favoring skeletal anchorage.

#### 3.8.1 Anchorage Loss: Horizontal Anchorage Loss in Upper Molars

Dental anchorage loss was a common side effect in the conventional treatment groups (Figure 6a). In contrast, the upper first molars in the skeletal anchorage groups showed significantly less horizontal movement with a mean difference of −1.14 mm (95% CI: −2.12 to −0.17 mm). Sar et al. reported the least mesial molar movement of −0.03 mm (SD 0.63) among the skeletal anchorage groups (Şar et al. 2011). Across all studies, the conventional treatment groups showed greater dental anchorage loss, ranging from 1.69 mm to 5.94 mm, while SA treatment groups showed less anchorage loss, ranging from −0.03 mm to 4.77 mm (Liang et al. 2021). There was low heterogeneity between the studies (*I* ^2^ = 10.3%, *Q* = 3.34, df = 3, *p* = 0.34).

#### 3.8.2 Anchorage Loss: Changes in Upper Molar Extrusion

The studies also evaluated the extrusion of the upper first molars, indicating the anchorage loss in the vertical dimension. The meta‐analysis results showed that molar extrusion was significantly less in the skeletal anchorage groups, with an MD of −0.74 mm (95% CI: −1.07 to −0.40 mm), ranging from 0.16 mm to 1.20 mm (Figure 6b). There was low heterogeneity between the studies in these groups (*I* ^2^ = 0.0%, *Q* = 1.15, df = 3, *p* = 0.76), suggesting consistency in the findings.

#### 3.8.3 Anchorage Loss: Changes in Maxillary Incisor Proclination

Four of the included studies assessed the changes in maxillary incisor proclination in relation to the palatal plane (U1‐PP), whereas two used the cranial base. Overall, incisor proclination changes were less common in the SA group (MD = −2.47°; 95% CI: −4.14° to −0.80°), with low heterogeneity across studies (*I* ^2^ = 0.0%, *Q* = 1.59, df = 3, *p* = 0.66) (Figure 6c). Aglarci et al. (2016) reported mean proclination changes of 3.01° (SD 4.14) in the skeletal anchorage group versus 5.90° (SD 3.68) in the conventional group. Sar et al. (2011) observed mean changes of −0.83° (SD 3.95) (skeletal anchorage) and 2.36° (SD 3.14) (conventional). Liang et al. (2021) reported a smaller difference between groups, 2.76° (SD 3.94) in the SA group, and 3.85° (SD 5.76) in the conventional group. In Alzoubi et al. (2023), upper incisor inclination increased in both groups (SA: 2.60° [SD 11.77], conventional: 3.20° [SD 6.53]).

#### 3.8.4 Changes in SNA Angle

Using the bone‐anchored maxillary protraction (BAMP) protocol, Aglarci et al. achieved an improvement in the SNA angle of 1.63° (SD 1.30) in the skeletal anchorage group, compared to 1.34° (SD 0.91) in the conventional facemask group (Ağlarcı et al. 2016). Sar et al. (2011) used a miniplate‐anchored facemask following RME and reported an SNA angle improvement of 2.53° (SD 1.24) in the skeletal anchorage group, while the conventional treatment group showed a smaller improvement of 1.83° (SD 1.33). The two other studies that also performed RME before bone‐anchored facemask treatment reported similar findings (Alzoubi et al. 2023; S. Liang et al. 2021). The remaining study using bone‐anchored maxillary protraction with a facemask showed an SNA angle improvement of 2.20° (SD 1.30), compared to 1.10° (SD 1.00) in the conventional treatment group (Seiryu et al. 2020). Overall, the random effects meta‐analysis confirmed a greater improvement in the SNA angle in the SA group, with a mean difference of 0.65° (95% CI: 0.18°–1.13°), in comparison to the conventional treatment group, with low heterogeneity between studies (*I* ^2^ = 0.0%, *Q* = 3.67, df= 4, *p* = 0.45) (Figure 6d). The funnel plot did not reveal any signs of publication bias.

#### 3.8.5 Changes in Mandibular Plane Angle (SN‐MnPl)

Four studies assessed the change in mandibular plane angle (SN‐MnPl). The pooled estimate showed that the SA group had a slightly less clockwise rotation, but the effect was small and not statistically significant (MD = −0.38°; 95% CI −1.67 to 0.91) (Figure 6e). Heterogeneity was moderate (*I* ^2^ = 45.6%, *Q* = 5.51, df = 3, *p* = 0.14), suggesting some variability between the studies. Aglarci et al. (2016) found significant mandibular clockwise rotation in both groups, but slightly more in the SA group (1.66° [SD 1.87] vs. 1.30° [SD 2.11]); Sar et al. (2011) showed posterior rotation in both groups with a larger effect in the FM group; Liang et al. (2021) reported minimal mandibular plane changes in the SA group; Seiryu et al. (2020) was the only study to show virtually no change in SN‐MnPl in either group. Overall, the random effects meta‐analysis results do not show a clinically significant difference in mandibular plane rotation between skeletal and conventional treatment groups.

#### 3.8.6 Changes in WITS Appraisal

Three of the included studies assessed the changes in WITS appraisal. The study by Aglarci et al. reported a greater WITS value improvement in the conventional treatment group (5.03 mm [SD 2.23]), in comparison to the skeletal anchorage group (3.87 mm [SD 2.55]) (Ağlarcı et al. 2016). Sar et al. found a greater WITS value correction in both groups, but no significant difference between groups, with 6.56 mm (SD 2.88) in the conventional group and 5.43 mm (SD 1.69) in the skeletal anchorage group (Şar et al. 2011). According to the estimation of means and standard deviation, Alzoubi et al. (2023) reported a slightly smaller WITS improvement in the conventional group (3.17 mm [SD 3.22]) compared with the skeletal anchorage group (3.33 mm [SD 2.86]). Overall, the random effects meta‐analysis results did not show a clinically significant difference between groups, that is, MD −0.88 mm (−2.49 to 0.73 mm). There was low heterogeneity between the studies (*I* ^2^ = 0.0%, *Q* = 1.25, df = 2, *p* = 0.54) (Figure 6f). Given the small number of studies and the wide confidence interval, the evidence is insufficient to determine whether a clinically relevant difference in WITS appraisal exists.

## 4 Discussion

The aim of this systematic review and meta‐analysis was to assess the efficacy of skeletal anchorage‐supported maxillary protraction compared with conventional facemask therapy, while controlling for the confounding effect of rapid maxillary expansion (RME), in growing patients with Class III malocclusion. Across the included studies, skeletal anchorage was associated with a modest, statistically significant greater forward movement of the maxilla (ΔSNA ≈+0.70°) and reduced dental side effects, including less molar mesialization (≈1.20 mm), less molar extrusion (≈0.70 mm), and less incisor proclination (≈2.50°) compared with conventional tooth‐borne protraction. These findings support that skeletal anchorage reduces unwanted dental compensations while maintaining or slightly enhancing the skeletal response.

Facemask protraction is typically recommended in early mixed dentition, when the circummaxillary sutures are not fully interdigitated, and the skeletal response is greatest (Baccetti et al. 1998). Skeletal anchorage may extend this treatment window into early adolescence (Kamath et al. 2022), although evidence remains limited. RME is often combined with protraction to reduce sutural resistance and potentially enhance maxillary protraction. While some studies report greater maxillary advancement after RME, (Baik 1995; Gautam et al. 2009; Jäger et al. 2001; Kilic et al. 2008; Kim et al. 1999; Liou 2005; Liou and Tsai 2005), randomized clinical trials have not consistently confirmed this (Tortop et al. 2007; Vaughn et al. 2005). To minimize this confounding factor, the present review included only studies in which expansion was applied uniformly across all participants, allowing a more robust evaluation of skeletal anchorage effects.

Previous systematic reviews have evaluated skeletal anchorage for maxillary protraction, but none differentiated between studies with uniform versus non‐uniform RME protocols (Cornelis et al. 2021; Feng et al. 2012; Major et al. 2012; Podda et al. 2025; Rodríguez de Guzmán‐Barrera et al. 2017; Rutili et al. 2023; Wang et al. 2022). While some systematic reviews, including network meta‐analyses, reported greater orthopedic effects and fewer dental side effects with skeletally‐anchored approaches (Feng et al. 2012; Major et al. 2012; Podda et al. 2025), others found no clear evidence of better outcomes despite effective Class III correction (Cornelis et al. 2021; Rodríguez de Guzmán‐Barrera et al. 2017; Rutili et al. 2023). Podda et al. (2025) compared a SA group with an RME + FM group and reported greater WITS and SNA gains in the SA group, without controlling for the confounding factor of RME. Restricting inclusion to studies with comparable RME protocols does not change the overall direction of previous evidence, but it does make the results more reliable. Because RME has been shown to independently affect the skeletal response to maxillary protraction, comparing only studies in which RME was applied similarly in both treatment groups removes this potential confounder, allowing any observed difference between skeletal and conventional anchorage to be attributed more confidently to the anchorage system itself rather than to unequal expansion between groups. Table S2 summarizes the overlap in primary studies and the methodological differences across these systematic reviews. Overall, the evidence remains limited by small sample sizes, potential sample overlap, heterogeneous protocols, and short follow‐up periods.

The present systematic review included five prospective clinical trials (3 RCTs, 2 non‐randomized [Ağlarcı et al. 2016; Alzoubi et al. 2023; Liang et al. 2021; Şar et al. 2011; Seiryu et al. 2020]), comparing skeletal‐anchorage in the maxilla, one also using TADs in both jaws (Ağlarcı et al. 2016), with a conventional tooth‐borne facemask group. Both qualitative and quantitative analyses were performed, with sample sizes ranging from 34 to 50 patients. In one study (Alzoubi et al. 2023), missing standard deviations were estimated from medians and interquartile ranges (Luo et al. 2018; Wan et al. 2014).

All but one study (Alzoubi et al. 2023) evaluated dental anchorage loss on the upper first molar. The meta‐analysis showed that SA resulted in significantly less anchorage loss than conventional facemask therapy, both horizontally (−1.14 mm) and vertically (−0.74 mm). Sar et al. reported minimal molar mesialization in the SA group, likely because miniplates transmitted traction forces directly to the maxilla and treatment duration was shorter (≈6.80 months) (Şar et al. 2011). In contrast, Liang et al. (2021) observed greater mesialization after a longer traction period (≈10.80 months). Aglarci et al. (2016) also found reduced mesialization and extrusion in the SA group, probably due to the use of bonded intraoral appliances, which helped control vertical changes.

These findings, consistent with previous reports (Maino et al. 2018; Nguyen et al. 2011), indicate that skeletal anchorage substantially limits undesired mesial drift and extrusion of the upper molars. Biomechanically, conventional anchorage transmits the reciprocal force through the periodontal ligament of the anchor teeth, producing mesial tipping and extrusion, whereas skeletal anchorage transmits force to the maxillary bone directly, with a shorter lever arm to the center of resistance, limiting dental side‐effects. The reduced horizontal and vertical changes indicate that SA enhances posterior anchorage, allowing protraction forces to act more effectively on the nasomaxillary complex while minimizing dental side effects. This mechanism theoretically promotes a more genuine orthopedic response.

Regarding side effects on the maxillary incisors, the meta‐analysis showed that skeletal anchorage significantly reduced incisor proclination compared with conventional facemask therapy, with a mean difference of −2.47°. Aglarci et al. (2016) reported the greatest difference, with nearly double the proclination in the conventional group (5.90° vs. 3.00°), possibly related to the use of a tooth‐borne acrylic splint. Sar et al. (2011) observed slight incisor retrusion in the miniplate group, suggesting that direct skeletal anchorage can almost eliminate dental side effects. Liang et al. (2021) also found greater proclination in the conventional group, though the initial incisor inclination was higher in that cohort. Alzoubi et al. (2023) reported similar findings, however, these results should be interpreted with caution, as the large standard deviations, derived from estimated quantiles, indicate high variability and lower precision in these values.

Because the anterior alveolar bone is mainly cancellous and therefore of limited anchorage quality (Rojo‐Sanchis et al. 2021), tooth‐borne traction transmitted through the periodontal ligament of the incisors produces labial tipping, particularly when mild dental compensation is already present. The observed difference may therefore be clinically relevant, particularly in patients with pre‐existing dental compensation. Interpretation, however, should consider baseline proclination differences among studies.

The dental side effects observed in the present systematic review are in line with previous studies, indicating that conventional facemask therapy is associated with maxillary mesial molar drift, molar extrusion and incisor proclination (Baik 1995; Kapust et al. 1998), effects that are unfavorable in Class III patients, who frequently exhibit dental compensation. Skeletal anchorage may help limit these effects by improving force transmission and reducing dental side effects (De Clerck et al. 2009; Kircelli and Pektas 2008).

All included studies reported changes in the SNA angle, consistently showing greater sagittal improvement with SA. This improvement may be related to more direct skeletal force application and reduced dental interference (De Clerck et al. 2009; Nienkemper et al. 2015). Studies combining SA with RME or Alt‐RAMEC tended to report larger SNA increases (Alzoubi et al. 2023; Liang et al. 2021; Şar et al. 2011), although the contribution of expansion cannot be clearly isolated. Aglarci et al. (2016) observed a slightly greater improvement in the SA group despite higher applied forces in the conventional group, possibly reflecting anchorage loss. The same study also showed the smallest overall SNA change, likely due to the older age of participants.

The meta‐analysis revealed a mean SNA difference of +0.65°, which was statistically significant but limited clinical relevance. A difference of 0.65° falls within the range of typical cephalometric method error for SNA (approximately 0.5°–1°), meaning that, for an individual patient, this change cannot reliably be distinguished from measurement error and is unlikely to produce a visible change in facial profile. This sagittal change should, therefore, be interpreted cautiously and not in isolation, as maxillary advancement is closely associated with concomitant vertical and rotational effects. The limited SNA increase may reflect a more controlled skeletal response with reduced dental compensation, rather than a lack of orthopedic effect. A comprehensive evaluation including both sagittal and vertical parameters is therefore necessary to better assess the clinical relevance of skeletal anchorage.

Four studies reported changes in the mandibular plane angle (SN‐MnPl) (Ağlarcı et al. 2016; Liang et al. 2021; Şar et al. 2011; Seiryu et al. 2020). Results showed slightly less clockwise rotation in the SA group (MD −0.38°), although the effect was small and not statistically significant. Aglarci et al. (2016) reported slightly greater clockwise rotation in the SA group, possibly related to bonded intraoral appliances controlling maxillary molar extrusion and mandibular plane rotation. Sar et al. (2011) observed greater rotation in the FM group. Placing miniplates closer to the center of resistance of the nasomaxillary complex may reduce anterior maxillary rotation and thereby limit mandibular plane changes.

In contrast, Seiryu et al. (2020) applied a nearly horizontal vector (< 3° to the occlusal plane), passing below the maxillary center of resistance, which likely induced a counterclockwise moment on the nasomaxillary complex. This resulted in anterior intrusion, posterior extrusion, and a mild increase in palatal plane angle, with reduced overbite, while SN‐MnPl remained largely unchanged. This indicates that vertical effects may occur primarily at the maxillary level. The combination of anterior intrusion and posterior extrusion likely caused anterior rotation of the palatal plane and mild downward displacement of posterior maxillary structures, reducing the effective forward displacement of A‐point and thereby limiting the SNA gain despite the high applied force. SA limits the molar extrusion that causes clockwise mandibular rotation in conventional facemask treatment, while its more posterior force application allows the force vector to be positioned closer to the maxillary center of resistance. These findings show that near‐horizontal force vectors applied below the maxillary center of resistance can induce unfavorable vertical effects (Mermigos et al. 1990; Tanne and Sakuda 1991).

In summary, SA may reduce clockwise mandibular rotation by limiting molar extrusion, but the pooled effect was small, heterogeneous, and not statistically significant. Interpretation is further limited by the lack of reported maxillary vertical parameters (SN‐NL), which limits a comprehensive assessment of the vertical impact of skeletal anchorage. Future analyses should consider the influence of force vector, anchorage type, RME use, treatment duration, and skeletal maturity.

In the random effects meta‐analysis of WITS appraisal measurements, which included three of the studies, the conventional treatment groups showed a slightly greater apparent improvement (MD = −0.88 mm) (Ağlarcı et al. 2016; Alzoubi et al. 2023; Şar et al. 2011). At first glance, this may challenge the assumption that SA results in superior anteroposterior correction. However, in Aglarci et al. (2016) the conventional group exhibited greater molar extrusion and a steeper occlusal plane angle, which likely contributed to an apparent WITS improvement without true sagittal skeletal change. As WITS depends on projections onto the functional occlusal plane, any clockwise rotation of that plane (bite opening) moves point A's projection anteriorly and point B's projection posteriorly, thereby increasing the WITS value even in the absence of true skeletal advancement. Consequently, the apparent improvement in the conventional groups likely reflects occlusal‐plane rotation and posterior extrusion rather than genuine sagittal advancement.

Sar et al. (2011) reported greater overall WITS correction, slightly favoring the conventional group, possibly due to prior RME. However, part of the observed improvement could reflect vertical dental effects rather than genuine skeletal change.

Therefore, the greater WITS improvement in conventional groups should be interpreted cautiously, as it may largely reflect occlusal plane rotation and posterior extrusion rather than enhanced orthopedic effect. While the pooled analysis suggests only modest skeletal differences, this does not imply that SA is clinically irrelevant, particularly in reducing anchorage loss. Although Nienkemper et al. (2015) also reported similar improvements with conventional FM/RME, Lee et al. (2020) found greater skeletal improvement with SA in young Class III patients. Evidence on age‐related effects remains limited, although sutural maturation likely influences treatment response.

### 4.1 Quality of Evidence

Robust controlled trials directly comparing RME or Alt‐RAMEC with and without SA, particularly in older children (≈12–14 years), remain limited. Some evidence suggests potential benefits of SA in this age group. For example, a multicenter RCT by Mandall et al. (2024) reported improved ANB with bone‐anchored maxillary protraction, and long‐term data by Meyns et al. (2025) indicated sustained skeletal correction following combined Alt‐RAMEC and SA protocols. A review by Kamath et al. (2022) further suggests that SA becomes more relevant as circummaxillary sutures begin to interdigitate. A recent case report indicated that a 16‐year‐old treated with Alt‐RAMEC and miniplates showed favorable outcomes (Büyükçavuş et al. 2023). However, these findings are based on a limited number of studies with heterogeneous designs and should be interpreted with caution.

Overall, the available evidence suggests that SA combined with expansion protocols may be beneficial in older children, particularly as sutural maturation progresses. Future research should include well‐designed, age‐stratified randomized trials with standardized protocols and long‐term follow‐up to assess the true orthopedic potential of SA once suture patency decreases.

### 4.2 Limitations

This meta‐analysis included only studies with uniform RME protocols, which reduced confounding but limited assessment of the independent effect of expansion. Variations such as RME versus Alt‐RAMEC may still have influenced outcomes and contributed to residual heterogeneity. Assessment of vertical changes was also limited, as most studies reported only SN‐MnPl, without evaluating maxillary rotation. A more comprehensive analysis including SN‐NL and ML‐NL angles would allow a clearer evaluation of vertical and rotational changes in both jaws.

One study required reconstruction of means and standard deviations from medians and interquartile ranges, which may add uncertainty (Alzoubi et al. 2023). The overall level of evidence remains limited by small sample sizes, heterogeneous treatment protocols, differences in treatment duration and force magnitude, varying age ranges, and short observation periods. In addition, non‐randomized studies presented a higher risk of bias, mainly related to confounding and selection, which may have influenced the results. Sensitivity analyses restricted to RCTs (Analysis A: with Alzoubi et al. 2023; Analysis B: excluding Alzoubi, given its reconstructed data) support this concern: none of the four poolable outcomes (anchorage loss, extrusion, SNA, SN‐MnPl), including three that were statistically significant in the primary analysis, remained significant once non‐randomized studies were excluded. Incisor proclination could be pooled only in Analysis A and was not significant, and WITS could not be pooled in either analysis due to sparse RCT reporting. With only 2–3 RCTs available per outcome, these analyses are themselves underpowered, and their results should be interpreted as hypothesis‐generating rather than definitive.

Furthermore, the small number of included studies limits the reliability of publication bias assessment. Although funnel plots did not show clear asymmetry, this cannot exclude small‐study effects or selective reporting. Funnel plot‐based assessments of publication bias are generally considered sufficiently informative only when at least 10 studies are available, whereas the present analysis included only five studies. For the same reason, the low *I* ^2^ values reported for some outcomes (df=1–3) should be interpreted cautiously, as they may reflect insufficient power to detect heterogeneity rather than true homogeneity.

Strict inclusion criteria also excluded studies comparing different skeletal anchorage systems (e.g., miniplates vs. palatal OMIs), further limiting the available evidence. However, clinical heterogeneity among the included studies should still be acknowledged, as the use of different anchorage protocols (miniplates, OMIs, hybrid expanders, palatal‐anchored systems, mandibular miniplates, intermaxillary elastics, and bone‐anchored facemask therapy) differs in biomechanics, anchorage location, and force application. These differences may have influenced the treatment outcomes and the interpretation of the pooled results.

Future research should prioritize well‐designed, adequately powered randomized trials with standardized protocols, age or skeletal‐maturity stratification, comprehensive vertical assessment, and long‐term follow‐up to better clarify the true orthopedic and dental effects of skeletal anchorage.

## 5 Conclusions

Within the limitations of the available low‐ to moderate‐certainty evidence, skeletal anchorage appears to reduce dentoalveolar side effects and provides modest improvements in skeletal outcomes compared with conventional facemask therapy. Larger, well‐designed randomized controlled trials with long‐term follow‐up are required to confirm these findings.

## Author Contributions

Conceptualization: Christianna Iris Papadopoulou, Nusha Paschaei, Rebecca Jungbauer, Giulia Brunello, and Kathrin Becker. Methodology: Christianna Iris Papadopoulou, Nusha Paschaei, Rebecca Jungbauer, Giulia Brunello, and Kathrin Becker. Data curation: Christianna Iris Papadopoulou and Nusha Paschaei. Investigation: Christianna Iris Papadopoulou and Nusha Paschaei. Formal Analysis: Christianna Iris Papadopoulou and Kathrin Becker. Validation: Kathrin Becker. Visualization: Christianna Iris Papadopoulou. Writing—original draft: Christianna Iris Papadopoulou. Writing—review and editing: Nusha Paschaei, Rebecca Jungbauer, Giulia Brunello, and Kathrin Becker. Supervision: Kathrin Becker and Giulia Brunello. Project Administration: Christianna Iris Papadopoulou. All authors read and approved the final manuscript.

## Funding

The authors have nothing to report.

## Conflicts of Interest

The authors declare no conflicts of interest.

## Supporting information


Supporting File 1

Supporting File 2

Supporting File 3

## Data Availability

The data extracted for the meta‐analysis are available in Table S1.
