# Supplementary material for: Comparison Between Skeletally‐Anchored and Conventional Maxillary Protraction in Growing Class ΙΙΙ Patients: A Systematic Review and Meta‐Analysis
Source: Clin Exp Dent Res. 2026 Sep 27;12(5):e70466. doi: 10.1002/cre2.70466 (PMC13616234; doi:10.1002/cre2.70466)
Supplement: Supplementary file 1 [file CRE2-12-e70466-s001.docx]

**Supplementary Table 2. Comparison of the present review with previous systematic reviews**

| **Review** | **Studies (n)** | **Search date** | **Skeletal finding** | **Dentoalveolar finding** | **Conclusion** |
| --- | --- | --- | --- | --- | --- |
| Present review | 5 (3 RCT, 2 CCT); RME matched | Jan 2026 | SNA +0.65–+0.70° (sig.); WITS n.s. | Less molar mesialization, extrusion, incisor proclination (sig.) | Modest skeletal gain, clearer dentoalveolar benefit with SA |
| Cornelis et al.  (Cornelis, Tepedino, Riis, Niu, & Cattaneo, 2021) | 28; RME not controlled | Jun 2019 | ANB +0.2° vs FM (n.s. clinically); WITS <1mm (n.s.) | Less incisor retroclination with BAC3E vs BAFM | Low overall evidence for BAMP skeletal effect |
| Feng et al.  (Feng et al., 2012) | 4; RME not controlled | Jul 2011 | +3.08mm advancement vs control; +1.41mm vs tooth-anchored | Less molar extrusion (−1.68mm), less proclination (−2.29°) | TAD-anchored MP may exceed tooth-anchored, but only 4 trials |
| Major et al.  (Major, Wong, Saltaji, Major, & Flores-Mir, 2012) | 2 clinical trials (+10 case reports); RME not controlled | Jun 2012 | SAMP +0.8mm A-N⊥F, +0.9° SNA vs DAMP (both sig.) | 3.2° less U1 proclination vs DAMP (n.s., P=0.075) | SAMP: same clinical endpoint, more skeletal change, low-fair evidence |
| Rodríguez de Guzmán-Barrera et al.  (Rodríguez de Guzmán-Barrera et al., 2017) | 9 (7 in meta-analysis); RME not controlled | Nov 2016 | WITS +1.28mm, SNA +0.60° vs facemask | Fewer dentoalveolar effects reported | Effective, but no clear superiority over traditional treatment |
| Rutili et al.  (Rutili et al., 2023) | 3 RCTs; RME not controlled | Jan 2022 | Greater SNA increase (n.s. clinically) | Less incisor inclination, more side-effects | Minor advantage; evidence very limited (3 RCTs) |
| Wang et al.  (Wang et al., 2022) | Network meta-analysis; RME not controlled | May 2021 | Greater SNA, ANB increase vs tooth-anchored | Less mandibular plane increase, less incisor proclination | Greater advancement, less rotation; mandibular growth unaffected |
| Podda et al.  (Podda et al., 2025) | 17 (5 RCT, 12 CCT); RME not controlled | Jul 2023 | ANB +2.511° vs +2.094°; WITS +4.691mm vs +3.781mm | Less U1-PP increase (+1.19° vs +5.62°), less overjet increase | Better skeletal correction, fewer side effects; long-term evidence limited |

n.s., not statistically significant; sig., statistically significant. None of the previous reviews restricted inclusion by RME/Alt-RAMEC protocol between compared groups.
