# Supplementary material for: Comparison Between Skeletally‐Anchored and Conventional Maxillary Protraction in Growing Class ΙΙΙ Patients: A Systematic Review and Meta‐Analysis
Source: Clin Exp Dent Res. 2026 Sep 27;12(5):e70466. doi: 10.1002/cre2.70466 (PMC13616234; doi:10.1002/cre2.70466)
Supplement: Supplementary file 2 [file CRE2-12-e70466-s003.docx]

**Supplementary Figure 1**. Random-effects meta-analyses including all available RCT data.

**Supplementary Figure 2**. Additional sensitivity analyses for outcomes affected by exclusion of Alzoubi et al. (2023).

**Supplementary Figure 3**. Funnel plots of included studies.
